# Legacy of the discovery of the T-cell receptor: 40 years of shaping basic immunology and translational work to develop novel therapies

**DOI:** 10.1038/s41423-024-01168-4

**Published:** 2024-05-31

**Authors:** Yufang Shi, Andreas Strasser, Douglas R. Green, Eicke Latz, Alberto Mantovani, Gerry Melino

**Affiliations:** 1 The Fourth Affiliated Hospital of Soochow University, Institutes for Translational Medicine, State Key Laboratory of Radiation Medicine and Protection, Medical College of Soochow University, Soochow University, Suzhou, 215000 China; 2 Walter and Eliza Hall Institute of Medical Research, Parkville, VIC 3052, Australia; Department of Medical Biology, The University of Melbourne, Melbourne, VIC 3010 Australia; 3 Department of Immunology, St. Jude Children’s Research Hospital, Memphis, TN 38105 USA; 4 Institute of Innate Immunity, University of Bonn, Bonn, 53127 Germany; 5 German Center for Neurodegenerative Diseases (DZNE), Bonn, 53175 Germany; 6 IRCCS Humanitas Research Hospital, Rozzano, MI Italy; 7 Department of Experimental Medicine, TOR, University of Rome Tor Vergata, 00133 Rome, Italy

**Keywords:** Immunology, T cells

Forty years have passed since the groundbreaking achievement of cloning T-cell receptor genes [1, 2]. The rich narrative that sets the stage for this significant event is incredibly striking, and its profound legacy in contemporary immunology and medical science is truly remarkable.

## Historical account

The Athenian historian Thucydides noted in 430 BC that those who had survived the plague in Athens were subsequently able to tend to the ill without fear of reinfection [3]. This concept of developing resistance following recovery from an infectious illness was later prominently confirmed in the smallpox pandemic. Notably, the practice of variolation was developed by Taoist alchemists who lived in solitude on Mount Omei in China around the beginning of the 10th century [4]. This technique spread via the Silk Road to Central Asia and Turkey and then to Europe, where it gained widespread acceptance, eventually leading Dutch physician Van Swieten to coin the term “immunity” in 1775 [5]. The concept of immunity rapidly gained traction in both medical circles and the general public, particularly after Edward Jenner’s discovery of vaccination using cowpox material. It was not until the 19th century, however, that immunology gained a solid scientific footing, with the realization that microorganisms were responsible for many infectious diseases and that the body possesses chemical and cellular defenses that can identify and eliminate foreign invaders, termed antigens [6].

## Appreciation of the specificity of immunity

The discovery of antisera in the late 19th and early 20th centuries revealed that the immune system could produce a vast array of different antibodies, each capable of targeting a specific antigen [6]. This phenomenon, known as the immune system’s diversity of specificities, highlights the body’s ability to counter a vast range of foreign substances or antigens, including but not limited to infectious pathogens. To account for this remarkable specificity and diversity, several theories were postulated. In 1900, Paul Ehrlich presented the side-chain theory, which suggests that cells are equipped with a variety of receptor side chains [7]. He posited that a vast array of antibodies already exist within the body prior to infection or challenge and that upon encountering an antigen, the corresponding antibody binds to it in a lock-and-key manner. This interaction was proposed to trigger the production of more antibodies with identical specificity. Linus Pauling, in 1940, proposed the template theory of antibody formation [8]. He argued against the notion of preexisting antibodies, instead proposing that antigens act as templates that shape the formation of antibodies, which then assume a structure complementary to the antigen, thus enabling precise binding. In the 1950s, Frank Macfarlane Burnet introduced the clonal selection theory, which hypothesized that each lymphocyte carries a unique antibody type [9]; this idea was then experimentally confirmed by Gustov Nossal and Joshua Lederberg [10]. When an antigen binds to an antibody, it prompts the lymphocyte to replicate itself, thereby creating a clone of cells with uniform specificity. This theory not only elucidated how a single antibody could be mass produced but also introduced the concept of immunological memory. Although these theories were not entirely accurate, they played a pivotal role as foundational elements in immunology, leading to an advanced understanding of how the immune system can create an extensive array of antibodies from limited genetic resources. In the early 1970s, at the Basel Institute for Immunology in Switzerland, Susumu Tonegawa built upon these concepts and demonstrated the genetic basis for the generation of antibody diversity via somatic recombination of Ig gene segments, which was not previously understood [11]. This groundbreaking discovery of the genetic mechanism responsible for the diversity of antibodies is known as V(D)J recombination of separate gene segments. Before his discovery, how the genetic code could contain enough unique antibody genes to match the virtually limitless array of antigens encountered by an organism was unclear. This discovery was a significant milestone in immunology and earned Tonegawa the Nobel Prize for Physiology or Medicine in 1987.

## The discovery of B cells and T cells

**Fig. 1:**
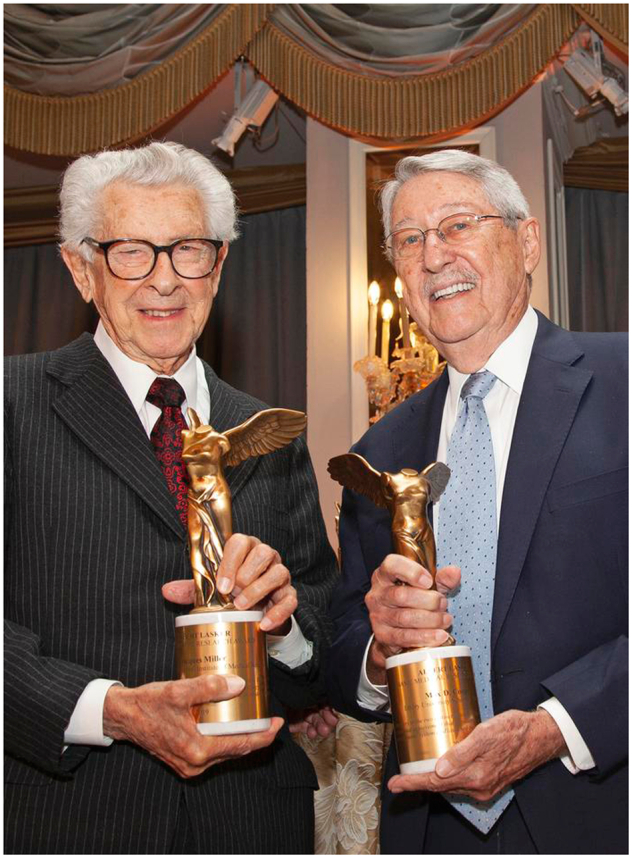
Jacques Miller and Max Cooper received the 2019 Lasker Basic Medical Research Award (Courtesy of Lasker Foundation)

As early as 1948, Astrid Fagraeus laid the groundwork for the fact that antibodies originate from plasma cells [12]. Subsequent research in 1956 by Bruce Glick and Timothy Chang revealed that B cells, which are produced in the bursa of Fabricius in birds and are so named “B cells”, are integral to antibody production, as evidenced by studies on chickens that had undergone bursectomy [13]. Between 1961 and 1962, Jacques Miller demonstrated that T cells, which develop from the thymus and are therefore named “T cells,” are crucial for the function of the adaptive immune system; Miller showed that thymectomy soon after birth rendered mice profoundly immunodeficient such that they could tolerate foreign skin grafts, even from rats, and they often died from infections [14]. Subsequent investigations, such as those using bone marrow chimeric mice with thymus grafts, by Jacques Miller and his student Graham Mitchell and by Max Cooper, revealed that thymus-derived lymphocytes, i.e., T cells, must cooperate with bone marrow-derived lymphocytes, i.e., B cells, so that robust antibody responses to immune challenge can be mounted [14, 15] (Fig. 1). These findings led to inquiries pertaining to the nature of antigen receptors on T cells.

## Conceptualization of the complexity of the TCR

In the dawn of research on the T-cell antigen receptor (TCR), the immunology community has grappled with many uncertainties about T-cell antigen recognition, MHC-restriction, avoidance of self-reactivity, and T-cell interactions with other cells. The enigma of how T cells discerned from non-self and recognized diverse antigens was particularly baffling. Without insight into these processes, understanding how T cells can effectively respond to pathogens without triggering autoimmunity was a challenge. The role of the MHC in antigen presentation is complex, as its exact influence on T-cell recognition was unclear; this lack of clarity made fully appreciating T-cell immune responses a challenge [16]. Moreover, preventing T cells from attacking the body’s own tissues—given the multitude of antigens they encounter—was a significant conundrum, with the mechanisms of self-tolerance and thus prevention of autoimmunity being crucial yet elusive. The nature of T-cell collaboration with other immune cells, particularly B cells, dendritic cells and macrophages, also remained mysterious.

At that time, researchers thought that T cells, similar to B cells, might be able to detect a diverse array of antigens through a clonally distinct recognition structure. This structure was thought to be similar (or, according to some studies, identical) to the immunoglobulins found in B cells. However, T cells are unique in that they require the presence of the appropriate major histocompatibility complex (MHC) molecules to bind antigens [17]. These MHC molecules were once targeted by subset-specific recognition elements known as T4 and T8, which are currently referred to as CD4 and CD8, respectively [18, 19]. Another molecule specific to the T-cell lineage, T3—a glycoprotein later named CD3ε—was discovered to be closely associated with T-cell functionality [20]. Ellis L. Reinherz and colleagues demonstrated that monoclonal antibodies against T3 (now called CD3) could impede certain T-cell activities, indicating that CD3 plays a role in the function of the antigen receptor [21]. As this protein is expressed nonclonally, there is a clear need to identify the clonally restricted component of the TCR that is specific to the antigen. This urgency and the maturation of technologies, such as monoclonal antibodies, T-cell hybridoma production, molecular biology, and, more importantly, the identification of the genes encoding antibodies, encouraged the search for TCRs. In 1981, Alan Harris and Jim Goding discovered a di-sulfide-bonded pair of proteins (i.e., proteins with characteristics similar to those of immunoglobulin on B cells) on T lymphoma cells [22], and in 1982, James P. Allison identified a clonally expressed T-cell surface epitope on murine T lymphoma cells [21]; these finding marked a pivotal moment in our understanding of the clonality of T-cell biology. Following these discoveries, in 1983, Ellis Reinherz made a significant contribution by defining the structure of the human T-cell receptor [23]. This elucidation was achieved using specific monoclonal antibodies against individual T-cell clones. This work was complemented by studies conducted in mice by Philippa Marrack and John Kappler, which provided valuable insights into the function of T-cell receptors [24]. These studies shed light on the complex interactions between T-cell receptors and antigens and further advance our knowledge of immune responses.

The abovementioned groundbreaking discoveries shed light on the existence of proteins with cellular clonality and revealed the potential for specificity to antigens, thus paving the way for further research in this critical area of immunology. One of the key contributions of these discoveries was the identification of the cellular clonality of T cells within the immune system [25]. The implications of these findings about T-cell clonality were profound, as they suggest that these cells may possess a high degree of specificity to antigens. However, the clonal size of the T-cell populations and the ways in which T cells interact with antigens through their antigen receptors were not known. Importantly, the genetic basis for the diversity of T-cell antigen receptors that recognize antigens urgently needed to be elucidated. This discovery would drive further innovations and breakthroughs in immunology, with far-reaching implications for human health and the treatment of diverse diseases.

## Genetic identification of the TCR

In August 1983, the Mark Davis group announced to the immunological community gathered at the International Congress of Immunology in Kyoto, Japan, that they had successfully isolated an mRNA encoding a component of the mouse TCR. An article describing the cloning of the TCR by Tak Wah Mak (Princess Margaret Cancer Centre, Toronto, Ontario, Canada) and one by Mark Davis and Stephen Hedrick (Stanford University School of Medicine, Stanford, CA, USA) appeared one after another on March 8, 1984 in Nature [1, 2]. Within a year, the alpha chain of the TCR was cloned, and the complete structure of the TCR was ultimately revealed [26–29]. In the same year, Tak Mak cloned the human TCR, described the rearrangements of genes encoding TCRs during T-cell maturation and neoplastic transformation, and published six articles in the journal Nature and three articles in the journal Cell [2, 30–37].

The approaches of Tak Mak and Mark Davis and their teams involved creating large T-cell cDNA libraries and employing subtractive hybridization with B-cell mRNA to isolate T-cell-specific sequences. Mak’s group successfully isolated a cDNA clone exclusive to T cells; sequencing revealed that this cDNA clone encoded a protein with a molecular weight of ~30 kDa [2]. This protein shared structural characteristics with the light chains of mouse and human antibodies. Davis and Hedrick, hypothesizing that TCR genes undergo rearrangement to engender diversity, used restriction enzyme analysis to differentiate their cDNA clones from genomic DNA in T and non-T cells [1]. They pinpointed a clone with a unique recombination pattern in T cells. They also detailed the sequence of this clone in the same publication, noting the presence of variable, constant, and joining segments that mirrored the structure of the antibody genes [38]. Further analysis confirmed that these cDNAs encoded the TCRβ chain [39]. A commentary by the well-respected immunologist Alan Williams included the statement, “The T-lymphocyte antigen receptor – elusive no more.” These pioneering findings paved the way for subsequent discoveries, including the various CD3 components and the TCRγ and TCRδ chains; these findings led to our current comprehension of the TCR complex as an eight-part receptor with intricate signaling pathways and functions.

**Fig. 2:**
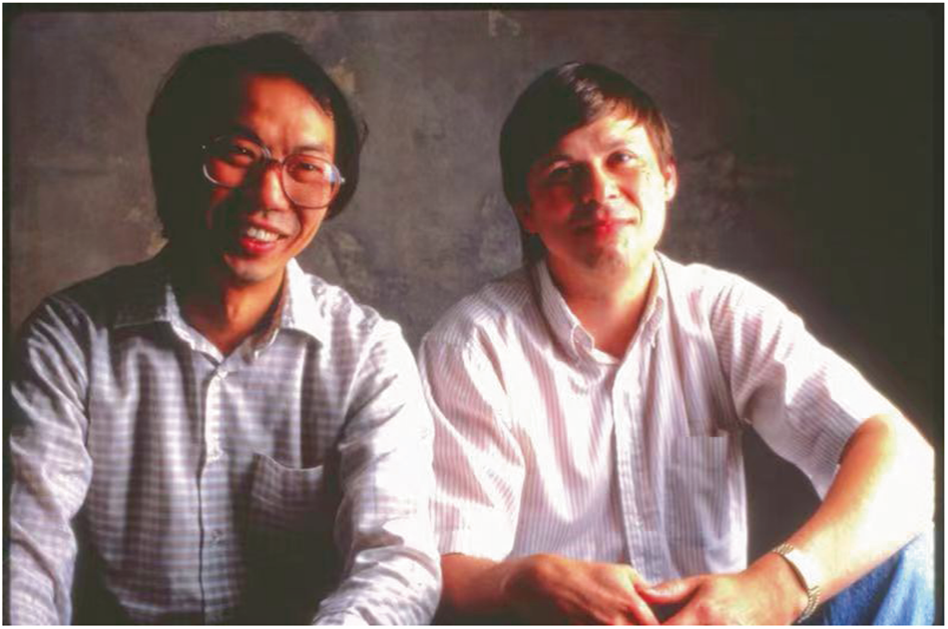
Tak Wha Mak and Mark Davis in 1984 (Courtesy of Gerry Melino)

This year marks the 40th anniversary of identification and cloning of the T-cell receptor (TCR). The search for the genes encoding the TCR, “the Holy Grail” of immunology, was a long and difficult task solved by two groups led by two young scientists, Tak Mak and Mark Devis (Fig. 2).

Their efforts allowed the immunological community to use cloned TCR molecular probes to examine known phenomena in T-cell biology and explore new hypotheses. The cloning of TCR genes also triggered an explosion of knowledge about the molecular structure of TCR proteins, the generation of TCR diversity, TCR expression in various T-cell subsets, and the involvement of the TCR in thymic repertoire selection and tolerance. In short, many questions that remained elusive due to the lack of molecular tools were answered quickly and emphatically. The discovery of the T-cell receptor allowed the immunology field to enter the molecular era [40]. Since then, TCR studies have continued to enrich our understanding of modern molecular medicine and immunology and shed light on the mechanisms underlying autoimmune diseases, infection control and cancer immunosurveillance. Indeed, the acquisition of new knowledge of TCRs and their functions continues today. In the 40 years since the first cloning of the TCR beta chain, more than 75,000 articles describing central and peripheral tolerance, molecular structure, peptide binding, structures of TCRs bound to MHC structures presenting antigenic peptides, and many clinical studies with therapeutic applications have been published on the TCR.

## The story of antigen recognition by TCRs

**Fig. 3:**
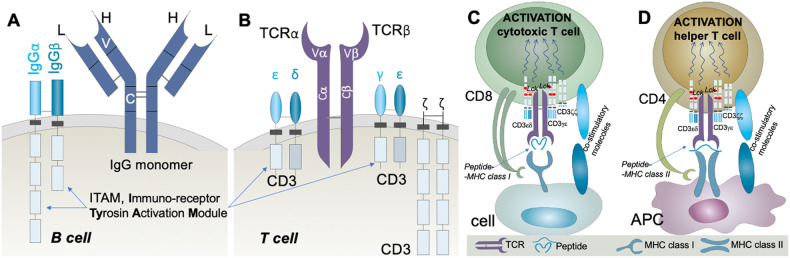
Structure and activation of the BCR and TCR complexes. **A** B cell antigen receptor complex; (**B**) T cell antigen receptor complex; (**C**) Interaction between a CD8^+^ T cell and a antigen presenting cell; (**D**) Interaction between a CD4^+^ T cell and a antigen presenting cell

Although they have the same ultimate function of eliminating non-self entities, T cells and B cells “see” their antigens in completely different ways. The B-cell receptor (BCR) is now known to be present on the surface of a B-cells as a transmembrane multiprotein complex that is composed of an immunoglobulin (Ig) (either IgM or IgD), which includes two identical heavy (H) chains and two identical light (L) chains, plus two accessory proteins called Igα (CD79A) and Igβ (CD79B) (Fig. 3A). The H and L chains of Ig combine to form two identical antigen binding sites that are capable of recognizing unprocessed antigen, while Igα and Igβ transmit intracellular signals that are triggered by antigen binding to the BCR [41, 42].

The BCR is capable of recognizing cell surfaces or soluble antigens from a wide range of chemical structures without the need for processing/modification. The TCR complex, which is positioned in the membrane of a T cell, is composed of a heterodimeric TCR receptor protein (αβ or γδ) and five accessory CD3 proteins (CD3γ, CD3δ, CD3ε, CD3ζ, and CD3η) that transmit activation signals intracellularly when the TCR is engaged [43, 44] (Fig. 3B). In most human and mouse T cells, the receptor protein is composed of a TCRα chain and a TCRβ chain, with only a minority of T lymphocytes carrying an antigen receptor composed of a TCRγ chain and a TCRδ chain. TCRα/β and TCRγ/δ T cells are thought to be activated by distinct ligands and involved in immune responses against different pathogens [45].

The intracellular portions of TCR chains are too short to signal; therefore, TCRs rely on the CD3 complex to transduce intracellular signals upon activation. The CD3 complex is composed of several different heterodimers and homodimers, including a CD3ε-CD3δ heterodimer and a CD3γ-CD3ε heterodimer [43, 44]. The most significant difference from BCRs is that TCRs do not bind to whole soluble molecules but recognize an antigen peptide when it is derived from and “presented” by another cell, called an antigen-presenting cell (APC). The initiation of T-cell responses requires a particular type of APC called a dendritic cell. The discovery of dendritic cells and their role in antigen presentation to T cells by Ralph Steinman in 2011 led to him being awarded a Nobel prize.

The presentation of fragments derived from antigens to TCRs on T cells is mediated by MHC proteins. This presentation to the TCR is mediated by the formation of a unique epitope (pMHC) that is composed of a peptide (p) derived from an antigen linked to an MHC protein complex that is expressed on the surface of another cell. Cytotoxic T cells bind to peptide class I MHC complexes that can be found on any cell in the body (Fig. 3C), while so-called helper T cells (Th) recognize peptide class II MHC complexes that are found only on specialized APCs, such as dendritic cells (Fig. 3D). Cytotoxic T cells express the CD8 coreceptor, which binds to a portion of class I MHC outside of its antigen presentation groove, thereby enhancing the TCRα/β interaction [46]. T helper cells express the CD4 coreceptor, which enhances the interaction of their TCRα/β with class II MHC. The activation of both cytotoxic T cells and T helper cells can be markedly increased by the engagement of several costimulatory molecules, such as CD80 and CD86, which are expressed on dendritic cells [47]. Conversely, both cytotoxic T cells and T helper cells also express inhibitory coreceptors, such as CTLA4 and PD1. Their engagement by cognate ligands, CD80 and CD86 or PDL1 and PDL2, respectively, attenuates T-cell activation [48].

## MHC restriction

The discovery that T-cell activation was dependent on the presentation of antigens by self-MHCs (class I or class II), called MHC restriction, was made by Rolf Zinkernagel and Peter Doherty in the 1970s [17]. Originally, two possible explanations were proposed for this “MHC restriction.” One explanation is that there are two receptors on T cells, one for MHC and the other for antigen, and T cells are activated only when both receptors are occupied. Another model suggested that a single T-cell receptor recognizes a combination of antigen and MHC. The conundrum lies in how a vast number of different antigens can bind to a given MHC protein. In 1987, Pamela Bjorkman, Jack Strominger and their team provided a solution by revealing the structure of the MHC by X-ray crystallography [49]. Their work demonstrated that the first and second domains of the α-chain of MHC HLA-A2 form a groove that binds and presents an antigen-derived peptide and that the TCRα/β recognizes the overall conformation of the peptide and the edges of the groove of the MHC, thereby elegantly solving the long-standing mystery.

When a pathogen invades the body, the body first alerts the leukocytes of the innate immune system, including dendritic cells. These cells carry receptors, such as Toll-like receptors, that recognize highly conserved structural patterns of molecules expressed on a wide range of pathogens. Phagocytosis and inflammation mediated by the innate immune response keep microorganisms in check, while dendritic cells process microbial proteins and present peptides derived from them within MHC complexes [50]. Only then can the T lymphocytes of the adaptive immune response be marshalled into the fight against the invading pathogen. In the presence of cytokines secreted by cells of the innate immune system, such as macrophages and dendritic cells, T helper cells that recognize their TCRα/β peptide MHC complexes on the surface of APCs become activated and then undergo extensive clonal expansion (proliferation) and differentiation into effector T helper cells. These T helper effector cells secrete cytokines and provide the intercellular signals necessary to promote B-cell activation in concert with stimulation of their BCR by antigen. Activated B cells undergo extensive clonal expansion and differentiation into plasma cells that secrete antibodies, which are capable of directly attacking invading microbes, or into memory B cells that can rapidly respond to rechallenge with the same pathogen at a later time. Cytokines produced by T helper effector cells also support the clonal expansion and functional maturation of cytolytic effector T cells (CTLs), which can recognize antigenic peptide class I MHC complexes on the surface of infected cells and kill the cells [51]. In the pathogen invasion scenario described above, peptides derived from microbial proteins that are presented by MHC cause the activation, proliferation and functional maturation of T cells so that they can effectively attack foreign invaders. However, an APC does not take note of the origin of the proteins it processes and presents. Consequently, most peptides presented on MHC from APCs to T cells are derived from host proteins; that is, the peptides will be “self” in nature [52]. Since T cells expressing TCRs that recognize MHC-presenting “self antigen”-derived peptides can lead to attacks on healthy host tissues, these cells are usually eliminated or silenced through mechanisms of central tolerance (thymic selection) or peripheral tolerance, respectively. These mechanisms rely on the avidity of a T-cell’s TCR for its pMHC epitope, such that the TCR plays an essential role in preventing autoimmunity and infectious diseases [53]. Indeed, both the understanding of autoimmune diseases and the possible approaches to prevent or mitigate them in the future depend on a clear understanding of the structure and function of the TCR (and the BCR).

## Engineered TCRs

In 1989, Dr. Zelig Eshhar (The Weizmann Institute, Israel) used TCR gene cloning to develop a new method for cancer therapy [54]. He designed a hybrid protein composed of a variable region of an antibody that recognizes a B lymphoma/leukemia-associated surface marker, such as CD19 or CD20, and fused it with part of a TCR (VHSp6-TNT). This chimeric antigen receptor (CAR-T) facilitated T-cell-mediated killing of lymphoma or leukemia cells expressing the marker. CAR-T-cell therapy has been approved for the treatment of numerous types of relapsed or refractory aggressive leukemias and lymphomas, and this treatment has saved the lives of thousands of cancer patients to date [55, 56]. Much work is currently underway to develop effective CAR-T-cell-based approaches for T-cell-mediated attack of solid cancers. Using the abovementioned ingenious approach, Zelig Eshhar engineered the specific activation and targeting of cytotoxic lymphocytes through CAR-T cells in 1993 [57]. Through the subsequent efforts of Dario Campana, Michel Sadelain, Carl June and others, immunotherapy using CAR-T cells has become one of the most promising and powerful weapons in the fight against cancer, especially hematological cancer. These and other clinical applications of immunology would not have been possible without the basic discoveries of the TCR [55, 56].

Of course, research continues and seeks new avenues. In 2021, Naoto Hirano, in collaboration with Tak Mak, developed new procedures to purify tumor-infiltrating lymphocytes (TILs) and select ex vivo TILs with very high affinity for antigenic peptides from cancer-derived peptides, which are presented by class II MHC proteins, that can be cloned and amplified in culture [58]. This technology has been applied to antigenic epitopes in melanoma. Moreover, Christian S. Hinrichs (National Cancer Institute, National Institutes of Health, Bethesda, MD, USA) applied this strategy to engineer TCRs against E7 virus antigen-derived peptides presented by MHC in HPV-associated metastatic cancer. Phase 1 and 2 clinical trials (https://classic.clinicaltrials.gov/ct2/show/NCT02858310) reported robust clinical responses in 12 patients. Thus, TCR engineering is opening up promising immunotherapeutic approaches for solid tumors.

## Unraveling immune tolerance and immune checkpoints

Along with the recognition of the fascinating immune response of our body, scientists quickly realized that there must also be potent mechanisms to prevent the immune system from overreacting and from reacting to self-antigens. Paul Ehrlich, a great German scientist, is well known for his establishment of many histological staining methods and the pioneering development of chemotherapy. In the late 19th and early 20th centuries, Ehrlich made significant contributions to our understanding of the immune system, including the proposal of the “side-chain theory” (see above). He also proposed that the immune system must be able to distinguish between “self” and “non-self” to mount an effective defense against pathogens while avoiding harmful attacks on the body’s own tissues, according to his concept of “horror autotoxicus” [59]. This concept was a radical departure from the prevailing understanding of the immune system at the time, which held that the body’s defenses were primarily directed against external pathogens such as bacteria and viruses, but profoundly implicated our understanding of autoimmune diseases, which occur when the immune system mistakenly attacks the body’s own cells and tissues. Therefore, the body must have a remarkable ability to distinguish between self-antigens and non-self antigens. This conceptual process was poorly understood until 1945, when Ray Owen reported that heterozygotic twin cattle sharing a common placenta and possessing two distinct populations of red blood cells could tolerate organs transplanted to each other [60]. This so-called neonatal tolerance was further experimentally developed by Sir Peter Medawar through the adaptive transfer of spleen cells from a different mouse strain into neonatal mice. This experiment revealed that exposure to foreign antigens during a critical period of development could lead to long-term tolerance and the acceptance of grafts from the same donor [61]. This work earned Medawar a Nobel prize in 1960 together with Sir Frank Marfarlane Burnet (see above for his clonal selection theory). These observations led to the concept of immune tolerance through clonal deletion, whereby self-reactive lymphocytes are eliminated during development to prevent autoimmunity. Research on apoptosis has provided insight into the molecular mechanisms of this process. Studies using TCR (or BCR) transgenic mice revealed that high-affinity ligation of TCRs (and BCRs) in immature T cells (and B cells) during their development in the thymus (or bone marrow) causes the induction of proapoptotic BIM (and some of its relatives, e.g., PUMA), which then kill these potentially dangerous lymphocytes [62, 63]. Accordingly, defects in apoptotic cell death in T and B lymphocytes caused severe autoimmune disease in mice [64, 65].

Discovery of the TCR prompted Harald von Boehmer and several others with their colleagues to generate TCR transgenic mice [66]. In one of the first TCR transgenic mouse experiments, researchers used a TCR isolated from T cells that could specifically recognize and kill APCs presented through their class I MHC (H2Db), a peptide from H-Y (a male antigen) [67]. In a syngeneic background (H2Db), H-Y-specific T cells could readily develop in female mice but not in male mice, thus demonstrating that negative selection—the elimination of self-reactive T cells during their development in the thymus—could play a crucial role in preventing autoimmune responses. Notably, in the allogeneic background, H-Y-specific T cells could not be found in either male or female mice, suggesting another important process in the thymus, positive selection; positive selection is fundamentally important for the development of a functional immune system. Both positive selection and negative selection during T-cell development were verified through the genetic manipulation of TCR and MHC genes by the teams of Tak Mak and many others [68].

The discovery of TCRs also led to the recognition of a special group of antigens, superantigens, which are defined as antigens that are reactive to CD4^+^ T cells, bind to specific Vβs, do not require processing and presentation, and are not MHC restricted but require MHC II from any haplotype. Importantly, the groups of Philippa Marrack, John Kappler and others have shown that in the presence of so-called “superantigens”, such as the LTR of MMTV, cells bearing TCRs with a particular Vβ are eliminated during their development in the thymus, thus verifying the concept of negative selection [69, 70]. The field of apoptosis research has provided insight into the molecular mechanisms of negative selection. Studies using TCR (or BCR) transgenic mice revealed that high-affinity ligation of TCRs (and BCRs) in immature T cells (and B cells) during their development in the thymus (or bone marrow) causes the induction of proapoptotic BIM (and some of its relatives, e.g., PUMA), which then causes the death of these potentially dangerous lymphocytes [62, 63]. Accordingly, defects in apoptotic cell death in T and B lymphocytes cause severe autoimmune disease in mice [64, 65]. Moreover, during their development in the thymus, immature thymocytes expressing TCRs that are not able to bind with a threshold affinity to self MHC complexes without positive selection because they undergo apoptosis [71], although the processes initiating this cell death are not yet known.

Upon recognition of specific antigens presented by dendritic cells, T cells undergo a process of activation that culminates in their proliferation and subsequent differentiation into effector cells. However, engagement of the TCR complex alone is insufficient to fully activate T cells, and the provision of costimulatory signals, such as those mediated by the interaction of CD28 on T cells and CD80 or CD86 on dendritic cells, is necessary for robust T-cell activation. CD28 signaling promotes the activation of TCR-induced intracellular signaling pathways for the activation of critical transcription factors, such as NF-kB [72]; the activation of these transcription factors increased the survival rate and proliferation of activated T cells via the increased production of cytokines and chemokines and the upregulated expression of costimulatory molecules. Notably, antibodies against the T-cell surface-expressed protein CTLA4, which was discovered by Pierre Golstein, James Allison and their colleagues, promoted TCR ligation-induced activation of T cells [73, 74]. When Tak Mak’s team knocked out the gene for CTLA-4, the mice developed severe autoimmune diseases; thus, these results demonstrated for the first time that CTLA4 delivers negative signals to T cells [75]. This finding was supported by the confirmation that antibodies against CTLA-4, unlike antibodies against CD28, are antagonists rather than agonizts. This process is believed to be critical for preventing T-cell overactivation. The discovery of the role of CTLA-4 in attenuating T-cell responses has profound implications for cancer therapy [76]. CTLA-4-blocking antibodies, such as ipilimumab, have been developed to inhibit the inhibitory signal of CTLA-4, thereby enhancing T-cell activation, proliferation and effector functions. Ipilimumab and other CTLA-4 inhibitors have revolutionized the treatment of advanced melanoma and certain other cancers, thus highlighting the importance of the balance between activating and inhibitory signals in T-cell regulation.

Subsequently, using the activation-induced cell death (AICD) system, in which strong TCR stimulation causes apoptosis in previously activated T cells (characterized by Jonathan Ashwell, David Lynch, Douglas Green, Yufang Shi, Peter Krammer, and others), Honjo, Arlene Sharpe and their colleagues discovered additional negative regulators of T-cell activation, such as programmed cell death protein 1 (PD1) and its ligands PDL1 and PDL2 [77–79]. Antibodies that block the functions of PD1 or PD-L1 have also become powerful for oncologists to combat cancer. The discovery of CTLA4 and PD1/PDL1 has not only transformed the landscape of cancer treatment but also revolutionized our understanding of immune regulation. These remarkable achievements in the field of immunotherapy were recognized with the Nobel Prize, which was awarded to James Allison and Tasuko Honjo. Their work and the foundational science it was based on continue to inspire ongoing basic research and translational developments in the field of immunology, with the promise of even more innovative treatments that can improve patient outcomes in the future.

## Other advancements in medicine emanating from the discovery of the TCR

In addition to the dramatic advancements in cancer patient care brought about by CAR-T, CAR-TCR, and immune checkpoint blockers, the discovery of the TCR has significantly advanced our understanding of the immune system, including the clonality of disease [80], and has also had a profound impact on vaccine development. TCRs are found on the surface of T cells and play a crucial role in recognizing and responding to foreign antigens, such as those present on invading pathogens, like viruses and bacteria. This recognition is a key step in the immune response, leading to the activation of T cells and the subsequent elimination of invading pathogens. The discovery of the TCR has provided valuable insights into how the immune system functions and has paved the way for the development of new and more effective vaccines. Understanding how T cells recognize and respond to antigens has been crucial for the design of vaccines that can effectively elicit T-cell-mediated immunity. For example, many vaccines are designed to elicit a strong T-cell response in addition to an antibody response to provide long-lasting protection against certain pathogens. This knowledge is being harnessed for the development of vaccines against infectious diseases, such as HIV, malaria, and tuberculosis, where traditional vaccine approaches have fallen short. By leveraging the precise targeting capabilities of engineered TCRs, researchers are exploring innovative vaccine strategies with the goal of inducing long-lasting immunity against persistent pathogens and attenuating aberrant immune responses in patients with autoimmune disorders.

In the future, the impact of TCR cloning on modern immunology will continue to reverberate. Ongoing research efforts are focused on further unraveling the complexities of T-cell recognition and activation, with the goal of refining existing immunotherapies, producing more potent (and safe) vaccines and developing novel treatment modalities for a myriad of diseases. The advent of next-generation sequencing technologies has enabled comprehensive profiling of TCR repertoires and provided unprecedented insight into the diversity and dynamics of T-cell responses in health and disease. Furthermore, advancements in genome editing techniques, such as CRISPR-Cas9, hold great potential for precisely engineering TCRs and enhancing their therapeutic impact and safety. The ability to manipulate TCR genes with great precision offers new opportunities for tailoring immunotherapies to individual patients and optimizing their effectiveness against a wide range of diseases.

In conclusion, the groundbreaking discovery by Tak Mak and Mark Davis of the genes encoding TCR T-cell antigen receptors has illuminated the field of modern immunology. This pivotal finding has transformed our understanding of T-cell development and function. Their pioneering work has not only deepened our knowledge of T-cell biology but also paved the way for the development of innovative immunotherapies that have revolutionized the treatment of various diseases. As we further decipher the complexities of TCR function and T-cell biology, the legacy of TCR cloning, as pioneered by Mak and Davis, will continue to influence the trajectory of scientific inquiry and innovation. New therapeutic interventions can be developed that are more effective, less invasive, and tailored to meet the individual needs of patients.
